# Immunity and Specific Therapy

**Published:** 1910-09

**Authors:** 


**Immunity and Specific Therapy.** By W. D'ESTE EMERY, M.D., B.Sc. Pp. xiv, 448. London: H. K. Lewis. 1909.

There was a need for this book, and Dr. Emery has met the requirements of the physician, surgeon and student in a manner demanding an expression of satisfaction and praise. Unlike that admirable volume on a similar subject recently issued by Professor Muir as a record of the sterling research work of the Glasgow School of Pathology, this book is written down to the limits of the general reader. Perhaps the best evidence of this fact is to be found in an admirable glossary which *precedes* the general text. This, at all events, allows the reader to spend a few hours of wonderment in the fairyland of bacteriological and serological terminology. A concise and explicit account of the history and general aspects of immunity is followed by chapters on the nature and interactions of toxins, the side-chain theory, bacteriolysins, agglutins, precipitins, phagocytosis, colloidal theories of antibodies, and the practical applications of immunity problems.

Much of what is written is now old, and the next edition will need considerable alteration, but it would be churlish to point out trivial defects, or dissent from the attitude of Dr. Emery on some contentious points. We express unreservedly our gratitude to Dr. Emery for the time and labour he has spent in compressing the subject within a few pages, and we hope that his efforts may meet with the success they deserve.

